# Neuromuscular Electrical Stimulation–Enhanced Physical Therapist Intervention for Functional Posterior Shoulder Instability (Type B1): A Multicenter Randomized Controlled Trial

**DOI:** 10.1093/ptj/pzad145

**Published:** 2023-10-23

**Authors:** Philipp Moroder, Katrin Karpinski, Doruk Akgün, Victor Danzinger, Christian Gerhardt, Thilo Patzer, Mark Tauber, Mathias Wellmann, Markus Scheibel, Pascal Boileau, Simon Lambert, Giuseppe Porcellini, Laurent Audige

**Affiliations:** Department of Shoulder and Elbow Surgery, Schulthess Clinic, Zurich, Switzerland; Department for Shoulder and Elbow Surgery, Charité - Centrum für Muskuloskeletale Chirurgie, Berlin, Germany; Department for Shoulder and Elbow Surgery, Charité - Centrum für Muskuloskeletale Chirurgie, Berlin, Germany; Department for Shoulder and Elbow Surgery, Charité - Centrum für Muskuloskeletale Chirurgie, Berlin, Germany; Department of Traumatology, Hand Surgery and Sports Medicine, ViDia Clinics Karlsruhe, Karlsruhe, Germany; Department of Orthopaedics and Trauma Surgery, Schoenklinik Düsseldorf, Düsseldorf, Germany; Deutsches Schulterzentrum, ATOS Klinik, Munich, Germany; Orthoprofis, Hanover, Germany; Department of Shoulder and Elbow Surgery, Schulthess Clinic, Zurich, Switzerland; Department for Shoulder and Elbow Surgery, Charité - Centrum für Muskuloskeletale Chirurgie, Berlin, Germany; Institute for Sports & Reconstructive Surgery, Groupe Kantys, Nice, France; Department of Trauma and Orthopedics, University College London Hospital NHS Foundation Trust, London, UK; Department of Orthopedics and Traumatology, University of Modena and Reggio Emilia, Modena, Italy; Department of Shoulder and Elbow Surgery, Schulthess Clinic, Zurich, Switzerland

**Keywords:** Functional Shoulder Instability, Motion-Activated NMES, Neuromuscular Electrical Stimulation, Physiotherapy, Posterior Shoulder Instability

## Abstract

**Objective:**

Functional posterior shoulder instability (FPSI) (type B1) is a severe type of instability, mainly in teenagers and young adults, that leads to loss of function, pain, and stigmatization among peers. An experimental nonsurgical treatment protocol based on neuromuscular electrical stimulation (NMES) showed very promising early results in the treatment of FPSI. The hypothesis of this study was that NMES-enhanced physical therapy leads to better outcomes than physical therapy alone as the current gold standard of treatment in patients with FPSI.

**Methods:**

In this multicenter randomized controlled trial, patients with FPSI were randomly allocated in a 1:1 ratio to either 6 weeks of physical therapy or 6 weeks of physical therapy with simultaneous motion-triggered NMES. Baseline scores as well as outcome scores at 6 weeks, 3 months, 6 months, and 12 months after the intervention were obtained. The predefined primary outcome of this trial was the Western Ontario Shoulder Instability Index (WOSI) at the 3-month time point.

**Results:**

Forty-nine patients were randomized and eligible for the trial. The group that received physical therapy with simultaneous motion-triggered NMES showed a significantly better main outcome measurement in terms of the 3-month WOSI score (64% [SD = 16%] vs 51% [SD = 24%]). Two-thirds of the patients from the physical therapist group crossed over to the group that received physical therapy with simultaneous motion-triggered NMES due to dissatisfaction after the 3-month follow-up and showed a significant increase in their WOSI score from 49% [SD = 8%] to 67% [SD = 24%]. The frequency of instability episodes showed a significant improvement in the group that received physical therapy with simultaneous motion-triggered NMES at the 3-month follow-up and beyond, while in the physical therapist group, no significant difference was observed.

**Conclusion:**

The current study shows that NMES-enhanced physical therapy led to statistically significant and clinically relevant improvement in outcomes in the treatment of FPSI compared to conventional physical therapy alone—from which even patients with prior unsatisfactory results after conventional physical therapy can benefit.

**Impact:**

Based on the results of this study, NMES-enhanced physical therapy is an effective new treatment option for FPSI, a severe type of shoulder instability. NMES-enhanced physical therapy should be preferred over conventional physical therapy for the treatment of patients with FPSI.

## Introduction

**Figure 1:**
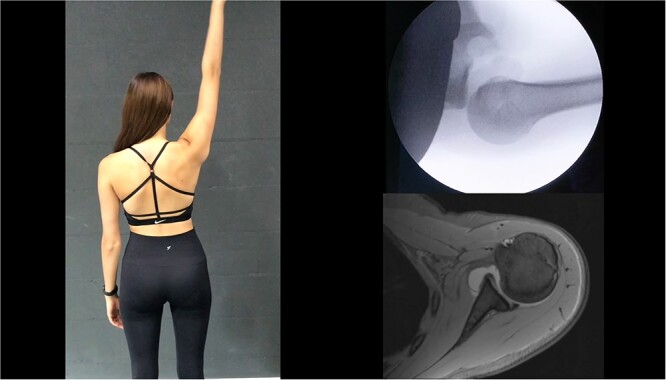
Patient with functional posterior shoulder instability (FPSI). The clinical image shows dynamic posterior subluxation of the humeral head during every elevation of the right arm, which can be visualized under fluoroscopic imaging. Despite this severe instability, no structural defects are visible on magnetic resonance imaging.

The interaction of active and passive restraints provides stability to the shoulder joint. As the active contraction of the shoulder surrounding muscles is key to maintain a centered joint,^1–4^ imbalanced muscle activation patterns can lead to a severe type of shoulder instability, recently named as “functional shoulder instability,” as opposed to its counterpart “structural shoulder instability,” which is caused by damage to passive anatomical restraints.^5^ The estimated prevalence of the often under- and misdiagnosed functional shoulder instability is 0.05% of the population^6^ and seems to be more common than expected, especially in young adults (0.5%–2.6%), with the peak of onset during adolescence.^7^ The most common type of functional instability is functional posterior shoulder instability (FPSI), which is caused by hypoactivity of external rotators and the posterior deltoid as well as hyperactivity of internal rotators and imbalance of periscapular muscles,^8^^,^^9^ leading to severe posterior instability occurring already in the midrange of motion despite the absence of critical structural defects.^5^^,^^10^ Affected patients may have a persistent feeling of instability as well as severely restricted function and recurring pain.^5^^,^^11^ Extensive limitations during daily and sporting activities as well as the “bizarre-looking dislocations” often lead to stigmatization and social exclusion from their peer group, with subsequent emotional distress of these young patients at a vulnerable age^5^ (Fig. 1).

Even though surgical treatment of patients with structural posterior shoulder instability has proven effective,^12^ its outcome in patients with FPSI is unpredictable. Instead of a desired stabilization effect, patients may experience increased pain, movement restriction, ticlike muscle cramping, as well as early glenohumeral degenerative changes.^9^^,^^13–16^ Therefore, the current gold standard treatment consists of nonsurgical interventions including core exercises, coordination training, strengthening exercises, as well as biofeedback training. These interventions, nevertheless, are not sufficiently effective in the treatment of FPSI in several patients.^9^^,^^15^ Skillful neglect has been proposed as an alternative treatment option as symptoms of FPSI seem to diminish with increasing age.^14^^,^^16^ However, a waiting approach is difficult to accept in the face of the severe symptoms experienced by patients with FPSI.^17^

After consultation with various shoulder specialists and several unsuccessful nonsurgical interventions, patients with FPSI often undergo unnecessary surgeries, which prove ineffective or actually worsen the condition.^5^^,^^17^^,^^18^ The limited effectiveness of available treatment options and the helplessness of shoulder specialists confronted with this pathology that cannot be identified on diagnostic imaging have even led to the dismissal of FPSI as attention-seeking behavior in the past, which further increased the disease burden and social stigmatization of patients with FPSI.^5^^,^^19^

An experimental nonsurgical treatment protocol based on neuromuscular electrical stimulation (NMES), named the shoulder pacemaker concept, showed promising results in the treatment of FPSI.^17^ The goal of this study was to evaluate the effectiveness and general applicability of this promising yet unproven treatment option for an unresolved clinical problem in a multicenter randomized controlled trial with independent data analysis and quality supervision by a study board of independent experts. The hypothesis of this study was that NMES-enhanced physical therapy leads to better patient-reported outcome than the current gold standard of treatment in patients with FPSI.

## Methods

The study was designed as a multicenter randomized controlled therapeutic trial with optional crossover, including 5 high-volume shoulder centers chosen for a balanced geographical distribution across a single country. Data storage, monitoring, and analysis were assigned to an independent center outside of the country. Additionally, an international independent expert board, including members from 3 different foreign countries, was established to supervise the study and assure adherence to the study protocol which was registered online prior to the beginning of the study including the prespecified outcome parameters in the ISRCTN registry (ISRCTN10085480). Ethical approval was obtained prior to the beginning of the study (Ethical Committee Charité University Hospital, Berlin, Germany; #EA2/077/18). The study was funded by the Deutsche Forschungsgemeinschaft that had no role in the design, conduct, or reporting of this study.

From January 2020 until September 2021, patients 13 years or older diagnosed with noncontrollable positional FPSI (type B1)^5^ were screened for exclusion criteria, including multidirectional instability, static posterior instability/head subluxation, connective tissue disease, degenerative joint disease, structural defects visible on pretreatment magnetic resonance imaging, neurological disorder or nerve injury, existing pain syndrome (defined by pain at rest or during motion, which is not caused by dislocation, but which impedes physical therapist training and/or NMES), contraindication to NMES treatment (eg, cardiac pacemaker), and previous participation in an FPSI-specific standardized NMES or physical therapist protocol. Due to request from different study sites, the following subspecification of structural defects visible on magnetic resonance imaging was added 2 months after the beginning of the trial, along with update of the online trial registration: (1) any acquired glenoid bone defect, (2) glenoid dysplasia with more than 10 degrees of retroversion of cartilaginous surface,^20^ (3) convex cartilaginous glenoid articular surface, (4) static posterior glenohumeral decentering of >55%,^21^^,^^22^ and (5) degenerative changes (any visible cartilage damage or osteoarthritis).

Patients fulfilling the eligibility criteria and with informed consent were enrolled by the local clinical investigator at each study site. After baseline assessment, patients were randomly allocated in a 1:1 ratio either to a 6-week physical therapist protocol or the same protocol with simultaneous motion-triggered NMES. The randomization sequence was stratified by site and based on random blocks of 2 and 4 as generated by the independent institution using Stata (StataCorp LLC, College Station, TX, USA) command “ralloc” and administered digitally within the study database in REDCap.^23^ Concealment of allocation before patient enrollment was preserved. The interventions were closely monitored for compliance and adherence to the protocol by the independent study supervision center by checking the mandatory treatment logs including number and date of treatments as well as achieved level per patient for each study site. A minimum attendance at 12 of the 18 training sessions was required to be considered a completed intervention. A longitudinal comparison of the predefined subjective and objective outcome parameters was performed at baseline, 6 weeks, 3 months, 6 months, and 12 months after the start of the intervention. An optional bidirectional crossover into the other intervention group (experimental or control) was possible after reaching the primary endpoint at the 3-month follow-up if the patient was not satisfied with the outcome.

The independent international advisory board received periodical recruitment and potential adverse events updates throughout the course of the trial and received access to the collected data at the end of the trial. Study reporting was strictly performed according to the Consolidated Standards of Reporting Trials guidelines.

### Interventions

The control intervention consisted of an FPSI-targeted physical therapist protocol with predefined exercises that were specified during a Delphi survey conducted among a panel of 9 scientific experts in the field.^24^ The protocol involved exercises with increasing difficulty and intensity. The experimental intervention consisted of the same physical therapist protocol but with additional simultaneous motion-triggered NMES stimulation of the external rotators and scapula retractors of the affected shoulder using rectangular symmetrically compensated alternate current with a frequency of 35 Hz and varying intensity based on the angle of motion of the arm and maximum intensity level set by the patient (Neuralign System S; Alyve Medical, Denver, CO, USA) (Suppl. Appendix). The stimulation frequency was predefined by the employed NMES device close to fusion frequency for upper extremity muscles and is intended to produce a subtetanic contraction while avoiding excessive muscle fatigue with the potential to induce supraspinal neural adaptations.^25–27^ Both interventions included 18 1-hour training sessions evenly distributed over a period of 6 weeks and were administered by the same physical therapist at each site. The physical therapists were instructed on the use of the device but did not have prior initial experience with the experimental intervention.

### Follow-Up Examinations and Study Parameters

The study protocol required a pretreatment assessment as well as follow-up evaluations at 6 weeks (end of treatment), 3 months, 6 months, and 12 months. Patients deciding for treatment crossover at 3 months were also assessed at 4.5 months. Patients were examined clinically and asked to complete various self-assessment questionnaires at these time points.

Patients were asked to complete the Western Ontario Shoulder Instability Index (WOSI), a shoulder instability-specific subjective outcome measurement that was the primary outcome parameter for this trial. It has proven high validity, reliability, and responsiveness^28^ and is recommended for the evaluation of shoulder instability.^29^ The reason to choose a subjective outcome measurement was the fact that the patients’ own perception of outcome was key to determining the success of the intervention,^30^ a valid, reliable, and comprehensive objective clinical or radiographic outcome parameter was not obtainable in this highly dynamic pathology,^17^ and objective clinical outcome measurements might vary slightly between participating centers despite standardization. The WOSI was inverted, ranging from 0 (worst outcome) to 100 (best outcome). Patients were also asked if they could dislocate their affected shoulder arbitrarily, or if it dislocated involuntarily in general (Likert scale: never, rarely, sometimes, often, always) and during activities of everyday life, work, or sports activity. Pain levels at rest and during motion on a numeric rating scale (NRS)^31^ and the Single Assessment Numeric Evaluation (SANE) score^32^^,^^33^ were also documented. Furthermore, patients were asked if their symptoms improved because of the treatment (yes/no), whether they would recommend their treatment to others (yes/no), whether they were satisfied with their treatment (on a numeric rating scale from 0 to 10), and whether they wanted to change to the other treatment arm of the trial due to dissatisfaction with the outcome at the 3-month time point. Active range-of-motion parameters of flexion, abduction, external and internal rotation at 0 degrees, and external and internal rotation at 90 degrees of abduction were evaluated. Muscle strength was recorded in flexion, abduction, and external and internal rotation using a portable manual dynamometer. Adverse events during and up to 12 months after the initiation of treatment were documented.

### Data Management and Statistical Analyses

Prior to the beginning of the trial, a required sample size of 66 patients was estimated based on an alpha error probability of 5%, a power of 80%, and an effect size of 0.8 and accounting for an expected dropout rate of 20% (13 patients) at 3 months. The effect size was calculated according to Cohen by dividing the minimal clinically important difference of the WOSI (10.4%)^28^^,^^34^ by the expected SD of the WOSI among the study participants (13%) based on available pilot data.^17^

Trial data were entered into the REDCap web-based Electronic Data Capture system,^23^ which was hosted on a dedicated password-protected server of an independent institution. Missing follow-up forms and data were closely monitored, and queries were generated back to the responsible study site for completion. Data were exported from REDCap into Stata software for statistical analyses.

Baseline characteristics were compared between the treatment groups by means of descriptive statistics and compared using standardized differences—calculated to 3 decimal places as the mean group difference divided by the common SD; values closest to 0.10 indicated stronger group similarity.^35^

First, the primary outcome parameter WOSI at 3 months was compared between treatment groups according to the study protocol using an independent-sample *t*-test after confirmation by a Shapiro–Wilk test that the parameter distribution did not significantly deviate from a normal distribution. The significance level was set at 0.05. The strength of effect is presented as the mean group difference along with its 95% CI. This efficacy analysis was performed on an intention-to-treat basis at the primary 3-month endpoint, which was also on a per-protocol basis given that patients not completing treatment withdrew their informed consent and could not be followed-up. We performed a sensitivity analysis using linear regression to control for potential confounding associated with observed differences in baseline WOSI, as well as the proportion of patients with both shoulder affected by the same pathology and the frequency of instability (after combining the categories “almost never” and “rarely”). The changes of WOSI from baseline to 3 months, separately for each treatment group, and from 3 months to 6 months in patients who crossed over from physical therapy to NMES-enhanced physical therapy, were examined with a paired *t*-test. To identify the characteristics of patients who benefited most from the NMES-enhanced physical therapy, baseline characteristics of patients with improvement above the minimal clinically important difference of the WOSI of 10.4% and those without the improvement were compared by means of nonparametric tests (2-sample Mann–Whitney test and Fisher exact test).

Secondary analyses compared treatment groups using unadjusted statistical testing (*t*-test and χ^2^ test for continuous and categorical outcome variables, respectively) regarding other outcome parameters documented at 3 months, including the SANE score, active range of motion, shoulder strength, pain level, frequency of instability episodes, patient’s ability to dislocate the affected shoulder arbitrarily, effect of shoulder discomfort on patient’s everyday activities, patient’s problems, and satisfaction level with treatment. The effect sizes were reported as the difference in group means for continuous parameters and relative risk for dichotomous outcomes, along with their respective 95% CIs.

Due to a large proportion of patients who crossed over from physical therapy to NMES-enhanced physical therapy, for graphical presentation of the outcome WOSI, SANE, and pain level, we considered initial physical therapist patients in 2 subgroups based on their treatment path after 3 months. The changes in all outcome parameters from the initiation of treatment to the final 12-month follow-up time point were explored using repeated-measures analyses separately by patient treatment groups and crossover subgroups.

The occurrence of adverse events was described.

### Role of the Funding Source

The study was funded by the Deutsche Forschungsgemeinschaft (MO-3414/1-1) that had no role in the design, conduct, or reporting of this study.

## Results

**Figure 2:**
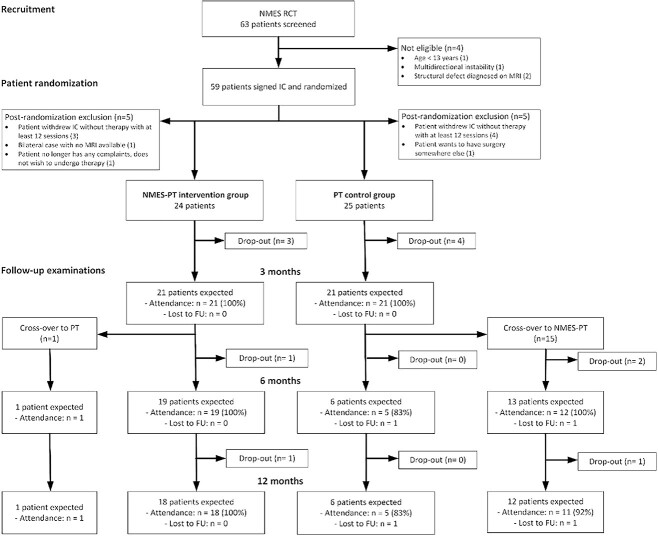
Study flowchart displaying patient recruitment, randomization, and follow-up process. FU = follow-up; IC = informed consent; MRI = magnetic resonance imaging; NMES-PT = neuromuscular electrical stimulation–enhanced physical therapy; RCT = randomized controlled trial; PT = physical therapy.

Patient recruitment proceeded as planned; however, toward the end of the trial period, a flattening of the recruitment curve was observed due to pandemic-related temporary closing of some study sites and the increased popularity and availability of the treatment concept at nonstudy sites slowing the number of referrals. Therefore, after counseling with the independent international study advisory board, recruitment was prematurely stopped after 59 patients instead of the originally planned 66. Ten patients had to be excluded after randomization due to ineligibility, 7 of whom lacked the required minimum completed training sessions. The follow-up rates were 85.7% for the primary endpoint at 3 months and 71.4% for the final follow-up at 1 year. Two-thirds of the patients from the physical therapist group crossed over to the NMES-enhanced physical therapist group due to dissatisfaction after the 3-month follow-up, and 1 patient crossed over from the NMES-enhanced physical therapist group into the physical therapist group (Fig. 2).

The comparison of baseline characteristics is displayed in Table 1. The standardized differences were low, except for a slightly higher frequency of bilaterally affected shoulders, instability, and more nonshoulder-related diseases in the NMES-enhanced physical therapist group. Both groups showed comparable adherence to the training sessions (*P* = .540).

**Table 1:** Comparison of Baseline Characteristics of Participants*^a^*

**Characteristic**	**NMES-Enhanced Physical Therapist Group (*n* = 24)**	**Physical Therapist Group (*n* = 25)**	**Standardized Difference** ^*b*^
Age, y, mean (SD)	19.8 (4.4)	20.1 (6.2)	0.061
Female sex assigned at birth	50	60	0.254
Dominant side affected	63	72	0.203
Body mass index, kg/m^2^, mean (SD)	22.6 (3.0)	22.7 (3.8)	0.022
Bilaterally affected	46	29	0.349
Frequency of instability			0.626
Almost never	0	8	
Rarely	8	20	
Sometimes	33	20	
Often	25	28	
Always	33	24	
Sulcus sign			0.133
Grade 0	38	44	
Grade 1	58	52	
Grade 2	4	4	
Beighton score			0.062
0–2 points	38	36	
3 or 4 points	33	32	
5–9 points	29	32	
Scapular dyskinesis	83	80	0.086
Other health problems	25	4	0.625
Western Ontario Shoulder Instability Index, mean (SD)	43 (17)	46 (19)	0.187
Single Assessment Numeric Evaluation score, mean (SD)	47 (22)	53 (19)	0.261

^a^ Data are reported as percentages unless otherwise indicated. NMES = neuromuscular electrical stimulation.

*^b^* The standardized difference was calculated to 3 decimal places as the absolute difference between group means divided by the common SD^36^; values closest to 0.10 indicated stronger group similarity.

**Figure 3:**
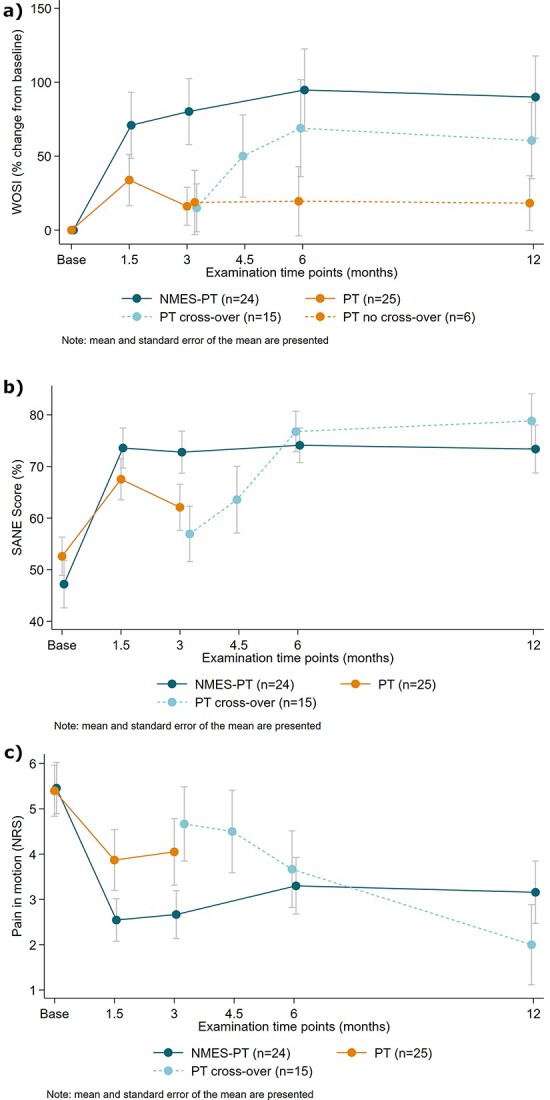
(a) Comparison of the change from baseline Western Ontario Shoulder Instability Index (WOSI) in the physical therapy (PT) and neuromuscular electrical stimulation–enhanced physical therapist (NMES-PT) groups, including crossover from PT to NMES-PT after 3 months. The mean and SE of the mean are presented. (b) Comparison of the Single Assessment Numeric Evaluation (SANE) score in the PT and NMES-PT groups, including crossover from PT to NMES-PT after 3 months. The mean and SE of the mean are presented. (c) Comparison of the pain level during motion in the PT and NMES-PT groups, including crossover from PT to NMES-PT after 3 months. The mean and SE of the mean are presented. NRS = numeric rating scale (0 = no pain and 10 = worst pain).

The NMES-enhanced physical therapist group showed a significantly better main outcome measurement in terms of the 3-month WOSI score (64% [SD = 16%]) than the physical therapist group (51% [SD = 24%]), with an absolute difference in means of 13% (95% CI = 1% to 25%; *P* = .047). Adjustment for baseline WOSI, as well as frequency of bilaterally affected shoulders and instability, resulted in a slightly stronger NMES-enhanced physical therapist effect, with absolute difference in means of 14% (95% CI = 1% to 28%; *P* = .036). The relative improvements compared to baseline were 80% (SD = 102%) in the NMES-enhanced physical therapist group and 16% (SD = 59%) in the physical therapist group (difference in means of 64%; 95% CI = 14% to 115%; *P* = .017) (Tab. 2). The improvement to 3 months was statistically significant and clinically relevant in the NMES-enhanced physical therapist group (mean change of = 21%; 95% CI = 12% to 31%; *P* < .001) but not in the physical therapist group (mean change of 4%; 95% CI = 6% to 14%; *P* = .418). The patients that crossed over from the physical therapist group to the NMES-enhanced physical therapist group showed a significant increase in the WOSI score from 49% (SD = 18%) to 67% (SD = 24%) (mean change of 17%; 95% CI = 3% to 31%; *P* = .023) at the 6-month follow-up (Fig. 3a). The patient that crossed over from the NMES-enhanced physical therapist group into the physical therapist group displayed no improvement in outcomes.

**Table 2:** Comparison of 3-Month Clinical and Patient-Reported Outcomes Between Groups*^a^*

**Outcome Parameter**	**Physical Therapist Group**		**NMES-Enhanced Physical Therapist Group**		**Effect Size (95% CI)** *^b^*	***P***
	***N* (%)**	**Mean (SD)**	***N* (%)**	**Mean (SD)**		
WOSI, %	21	51 (24)	21	64 (16)	13 (1 to 25)	.047
WOSI change, % of baseline value	21	16 (59)	21	80 (102)	64 (14 to 115)	.017
SANE score, %	21	62 (20)	21	73 (19)	11 (−1 to 23)	.085
Active range of motion, °						
Flexion	21	154 (42)	21	162 (30)	8 (−14 to 29)	.498
Abduction	21	146 (45)	21	157 (35)	11 (−13 to 35)	.384
External rotation in 0° abduction	21	77 (11)	21	81 (10)	5 (−2 to 11)	.155
Internal rotation in 0° abduction	21	82 (16)	21	81 (16)	−1 (−11 to 9)	.812
Shoulder strength, kg						
Abduction strength at 90°	16	6.2 (3.6)	16	6.5 (3.8)	0.3 (−2.3 to 2.9)	.833
Flexion strength at 90°	16	5.7 (3.1)	16	6.4 (3.7)	0.7 (−1.7 to 3.0)	.593
External rotation strength, seated	16	6.2 (3.0)	16	6.8 (3.2)	0.6 (−1.6 to 2.7)	.601
Internal rotation strength, seated	16	6.9 (3.3)	16	8.0 (4.4)	1.1 (−1.6 to 3.8)	.435
Pain level, NRS						
At rest	20	1.9 (2.5)	21	1.1 (2.0)	−0.8 (−2.2 to 0.6)	.260
During movement	20	4.0 (3.3)	21	2.7 (2.4)	−1.4 (−3.1 to 0.4)	.131
Patient can dislocate the affected shoulder arbitrarily						
No	1 (5)		4 (19)			
Yes	19 (95)		17 (81)		0.85 (0.68 to 1.1)	.174
How much does the shoulder discomfort hinder ..., NRS						
The patient’s everyday life?	21	4.4 (2.8)	21	3.1 (2.2)	−1.3 (−2.8 to 0.2)	.106
The patient’s work?	21	3.6 (2.9)	21	2.0 (2.1)	−1.6 (−3.2 to −0.1)	.045
The patient’s sports activities?	21	5.9 (2.7)	20	4.3 (2.7)	−1.6 (−3.2 to 0.1)	.077
Patient’s problems improved following treatment						
No	7 (37)		1 (5)			
Yes	12 (63)		20 (95)		1.5 (1.1 to 2.2)	.024
Patient’s level of satisfaction with treatment, NRS	20	6.8 (2.3)	21	8.4 (1.7)	1.6 (0.3 to 2.8)	.017

^a^ NMES = neuromuscular electrical stimulation; NRS = numeric rating scale, from 0 (minimum) to 10 (maximum); SANE = Single Assessment Numeric Evaluation; WOSI = Western Ontario Shoulder Instability Index.

*^b^* Effect size = difference in means with 95% CIs for continuous parameters and relative risk with 95% CIs for dichotomous outcomes.

Patients in the NMES-enhanced physical therapist group who showed clinically relevant improvement above the minimal clinically important difference of 10.4% had significantly lower baseline WOSI scores (mean = 37% [SD = 15%] vs 51% [SD = 16%]; difference in means of 14%; 95% CI = 2% to 27%; *P* = .043). An analysis of the influence of other preoperative factors on the treatment effect of NMES-enhanced physical therapy is shown in the Supplementary Appendix.

No significant differences in terms of the SANE score and pain at rest or during motion were observed between groups at the 3-month follow-up. Patients with crossover from the physical therapist group into the NMES-enhanced physical therapist group showed a significant improvement of their SANE score of 14% on average (95% CI = 2% to 25%; *P* = .027) (Fig. 3b and c).

**Figure 4:**
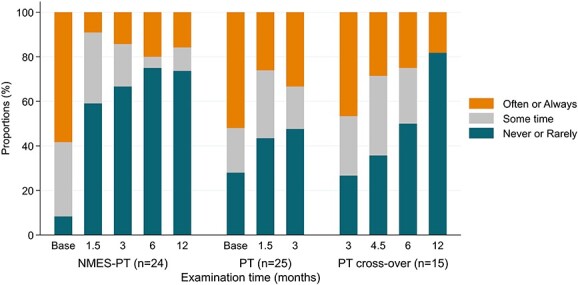
Frequency of instability episodes in the physical therapy (PT) and neuromuscular electrical stimulation–enhanced physical therapist (NMES-PT) groups, including crossover from PT to NMES-PT after 3 months.

The frequency of instability episodes showed a significant improvement in the NMES-enhanced physical therapist group at the 3-month follow-up (*P* = .001) and beyond (6 months: *P* = .001; 12 months: *P* = .004), while in the physical therapist group no statistically significant difference was observed (*P* = .481) (Fig. 4).

In both groups, most patients continued to be able to dislocate their shoulder voluntarily. Patients in the NMES-enhanced physical therapist group reported a subjective improvement of their symptoms in 95% of the cases, while patients in the physical therapist group reported an improvement in 63% of the cases (*P* = .024). The satisfaction level was significantly higher in the NMES-enhanced physical therapist group than in the physical therapist group (*P* = .017). Almost all patients in the respective groups would recommend their treatment to others (NMES-enhanced physical therapy: 100%; physical therapy: 90%). No differences in range of motion or in shoulder strength were detected (Tab. 2).

No adverse events other than recurrence of instability were recorded in either treatment group, except for 1 case of increased night pain and 1 case of depressive disorder in the physical therapist group.

## Discussion

We implemented a multicenter randomized trial that demonstrated a superior treatment effect of NMES-enhanced physical therapy over physical therapy in patients with FPSI when considering the predefined primary outcome measure (WOSI). Although NMES-enhanced physical therapy showed a statistically significant improvement from baseline exceeding the minimal clinically relevant difference at all follow-up time points, physical therapy showed no clinically relevant or statistically significant improvement from baseline. Moreover, even patients with secondary crossover to NMES-enhanced physical therapy due to unsatisfactory results after physical therapy showed a significant improvement further underlining the effectiveness of the shoulder pacemaker treatment concept.

Both the “muscular” and “neural” effects of NMES can offer a potential explanation for the improved outcome after NMES-enhanced physical therapy. It has been shown that NMES is able to improve force production and muscle volume of the infraspinatus,^26^^,^^36^ which has been identified as hypoactive in a dynamic fine-wire electromyography analysis of atraumatic posterior shoulder instability cases.^8^ However, no patients with muscle atrophies visible on magnetic resonance imaging demanding increase in muscle volume were involved in the trial and no shoulder strength differences between groups were detected at baseline or follow-up. An alternative explanation is neuroplasticity, the ability of the brain’s neural networks to grow and reorganize as part of a learning process. Howard et al discovered that patients with functional shoulder instability show an altered brain activity pattern similar to the early stage of learning a new motor sequence.^37^ Therefore, the cause of FPSI might rather be found in the brain activation pattern than in the periphery. The motion-triggered NMES utilized for this trial can be considered functional electrical stimulation, a subtype of NMES, in which the stimulation is paired to and assists purposeful movements, a concept which is widely used in patients with stroke.^38^^,^^39^ The coupling mechanism is the motion-triggered stimulation intended to improve timing of muscle activation patterns. A repeated, simultaneous presence of patients’ movement and NMES can induce neuroplastic changes that can improve motor function even after the treatment has ended.^38^^,^^40^ The applied low-frequency stimulation has the potential to induce supraspinal neural adaptations which yield improved motor control for novel tasks likely through increased corticospinal drive.^25^^,^^41^

However, it has to be mentioned that despite the superior outcome after NMES-enhanced physical therapy compared to control, the outcome scores after 1 year in terms of the WOSI (64% [SD = 20%]) and SANE (73% [SD = 20%]) are slightly lower than the mean values achieved in the first reports for the same treatment concept (WOSI: 70% [SD = 19%]; SANE: 81% [SD = 18%]). A possible explanation is that in contrast to the original trial the physical therapists of the various study sites involved in this trial were experienced with conventional physical therapy of shoulder instability but had no initial experience in the application of the new treatment concept. Therefore, the learning curve is included in the outcomes. Moreover, patients only had to complete 12 of 18 treatment sessions to be included in this trial, which is less than in the former trial. Therefore, this study assesses the effectiveness of NMES-enhanced physical therapy, in contrast to the focus on efficacy in the previous report. Additionally, the confirmation bias, which the original study has likely been subject to, was minimized by improvement of the study design of the present trial which also could explain the slightly worse outcome. Interestingly, the SD (±16%) and range (28%–89%) of the primary outcome parameter in this trial were quite large, indicating that some patients had a great benefit from the treatment while others did not. Although younger age, lower weight, unilateral affection, athletic activity have been identified as factors associated with better responsiveness to NMES-enhanced physical therapist treatment in the past,^17^ the subgroup analysis between patients with and without clinically relevant improvement of their WOSI score in this study showed no statistically significant differences. Nonetheless, the lack of statistical significance can be explained by the limited sample size in the subgroup and certain factors including younger age, female sex assigned at birth, lower weight, and unilateral affection seem to be worth to be further explored.

Additionally, it must be pointed out that treatment of patients with FPSI at best is able to convert the previously noncontrollable instability into a controllable condition. Therefore, patients will still be able to dislocate their shoulders at will after successful treatment.^17^ Furthermore, even in a patient with what subjectively would be considered a successful treatment occasionally a recurrent instability event might occur if the patient fails to concentrate and execute the correct muscle activation patterns. Therefore, success of treatment for FPSI cannot be evaluated on a binary basis of stable or unstable shoulder but rather must be interpreted on the basis of a more detailed grading scale of stability and subjective satisfaction of the patients. Accordingly, even though most patients improved their shoulder stability with the help of NMES-enhanced physical therapy performed primarily or secondarily after failed physical therapy, several still continued experiencing the occasional instability event after treatment as shown in Figure 4. In the physical therapist group, no significant improvement of the instability pattern was achieved. Accordingly, the rate of subjective improvement after treatment and satisfaction rate was higher in the NMES-enhanced physical therapist group. Nonetheless, almost all patients would recommend their respective treatments to others indicating their appreciation for both types of training sessions.

### Limitations

The inclusion and exclusion criteria were chosen to create a homogeneous group of study participants that still represents the typical characteristics of the majority of patients with FPSI. Despite the relative rareness of the pathology, the multicenter design including several referral centers allowed us to perform a randomized controlled trial with a reasonable power of 70% considering the initially expected effect size of 0.8, even though recruitment was prematurely stopped after 59 instead of 66 patients. Moreover, the multicenter design and the fact that physical therapists lacked initial experience for the investigated new treatment increased the external validity of the study results.

Comparability analysis of baseline characteristics between groups revealed low standardized difference values reassuring the success of the randomization process except for a slightly higher frequency of bilaterally affected shoulders, instability, and concomitant nonshoulder-related health conditions in the NMES-enhanced physical therapist group. However, statistical adjustment for potential confounding effect associated with observed minor baseline imbalances reinforced the study results, which show better outcome in the NMES-enhanced physical therapist group.

Adherence to the treatment protocol was not achieved in all patients, as in both groups some patients failed to complete the minimum amount of required 12 training sessions. This can partly be explained by the fact that the intervention consisted of 18 training sessions, and despite the strategic geographical choice of study centers across the country, several patients and their parents had to carry the burden of time-consuming frequent travel or costly hotel stays for the entire duration of the treatment. Moreover, motivational issues were sometimes encountered in this young patient cohort.

The reason for presetting the main endpoint at 3 months before the beginning of the trial was to ensure that the primary endpoint still can be obtained and only secondary endpoints will be jeopardized if a high rate of crossover from 1 intervention group to the other occurs. Crossover was allowed in the trial since, in the case of an unsatisfactory outcome after 3 months, no further improvement can be expected, thus warranting the offer of an alternative treatment to patients with FPSI. A reasonable follow-up period of 1 year was chosen, as the investigated interventions are noninvasive and can be repeated in case of deterioration of outcome over time.^17^ The achieved follow-up rate for the primary outcome measurement was 85.7% at 3 months and 71.4% at 1 year (final follow-up). Therefore, attrition bias cannot be excluded.

Since the primary outcome measurement was a patient-reported subjective score, the lack of blinding of the clinical examiner had limited consequences for the main study results. Blinding of the patients themselves was not possible due to the nature of the experimental and control interventions. This circumstance potentially introduced a confirmation bias from the patients’ side. However, according to the trial design, all patients with previous participation in a pathology-targeted standardized NMES or physical therapist protocol were excluded which reduced the risk for a preconditioned mindset in patients.

### Conclusion

The study results challenge the current treatment paradigms for FPSI. NMES-enhanced physical therapy leads to statistically significant and clinically relevant improvement of outcomes in the treatment of FPSI compared to conventional physical therapy alone. Even patients with prior unsatisfactory results after conventional physical therapy show a significant benefit after secondary NMES-enhanced physical therapy. Based on the results of this study, NMES-enhanced physical therapy should be preferred over conventional physical therapy for the treatment of patients with FPSI.

## Supplementary Material

2022-0742_R2_Supplementary_Appendix_TSR_pzad145

## Data Availability

Original study data is available upon request.
