# Supplementary material for: Neuromuscular Electrical Stimulation–Enhanced Physical Therapist Intervention for Functional Posterior Shoulder Instability (Type B1): A Multicenter Randomized Controlled Trial
Source: Phys Ther. 2023 Oct 23;104(1):pzad145. doi: 10.1093/ptj/pzad145 (PMC10824628; doi:10.1093/ptj/pzad145)
Supplement: 2022-0742_R2_Supplementary_Appendix_TSR_pzad145 [file 2022-0742_r2_supplementary_appendix_tsr_pzad145.pdf]

Training protocol for the trial:

Neuromuscular electrical stimulation enhanced physiotherapy for treatment of functional posterior shoulder instability- a multicenter, randomized controlled trial

General information

- 3 sessions of treatment per week over a period of six weeks
- 1 session of treatment = 60 minutes regular exercises
- Exercises under therapeutic supervision focusing on external rotators and periscapular muscles in patients with functional posterior shoulder instability
- Patient-specific, iterative increase of the difficulty and exercise complexity during the training (Level I-III)
- Milestones:
    - Level I: Regain ROM and functional coordination, decrease pain of the shoulder and minimize the occurrence of instability events
    - Level II: Improve strength, coordination and proprioception (mild to moderate resistance, high repetition), no pain and full functional ROM established
    - Level III: Further strengthen and increase full dynamic shoulder function, return to patient oriented sports training

Experimental intervention:

Additional application of neuromuscular electrical stimulation

(Neuralign System S, Alyve Medical)

- The NMES device remains attached to the shoulder to stimulate hypoactive muscle groups during all exercises.
- Prior to the physiotherapeutic treatment, the required intensity of the electric current is specified. It is important to use the highest current intensity the patient can comfortably handle (it typically increases over the course of the treatment).
- The electrode is positioned in the area of the nerve supplying the hypoactive muscle. Simultaneous stimulation of agonist and antagonist is to be avoided because of the resulting movement restriction. One electrode is placed inferior to the spina scapulae to stimulate the external rotators (M. infraspinatus, M. teres minor). The second electrode is placed medially to the margo medialis scapulae to stimulate the scapula retractors (M. trapezius, M. rhomboidei).
- Aim for an initial abduction-external rotation of the arm due to the electrical stimulation of external rotators and scapula retractors. If sharp pain occurs slightly vary the electrode positioning or decrease the stimulation energy for comfortable training.

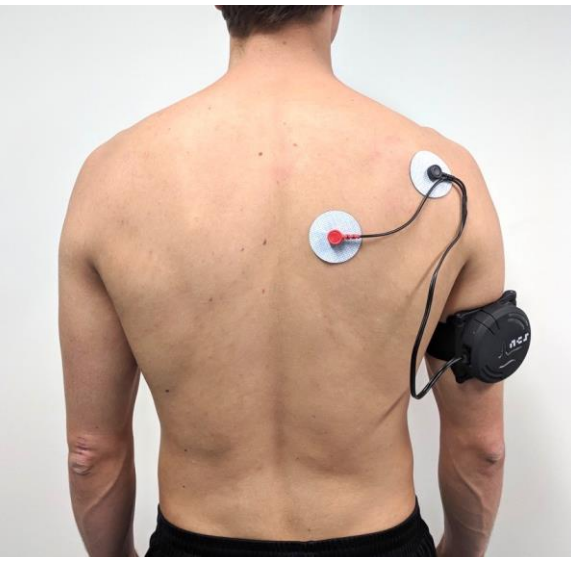
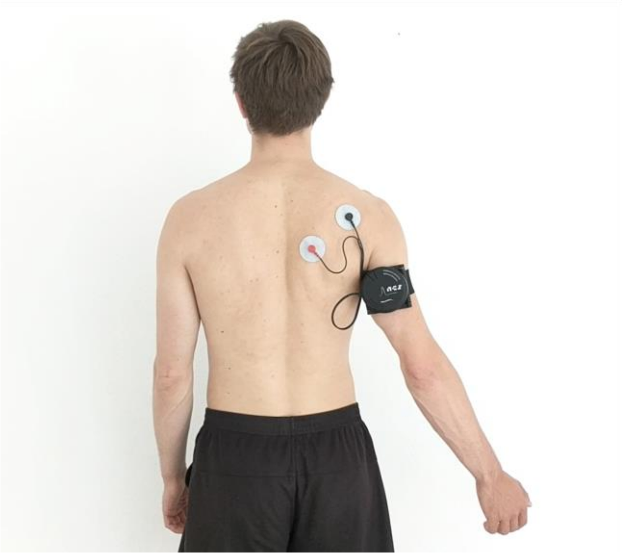

1. **Exercise program**

Level I:

Exercise 1.1: Elevation with lateral resistance ("Lateral wall slide")

- Starting position: Patient with the affected arm against a wall
- Exercise: Elevation, back of the hand facing the wall, 3x15- 20 repetitions
- Note: Start to 90° Flexion, slowly progress for pain free motion

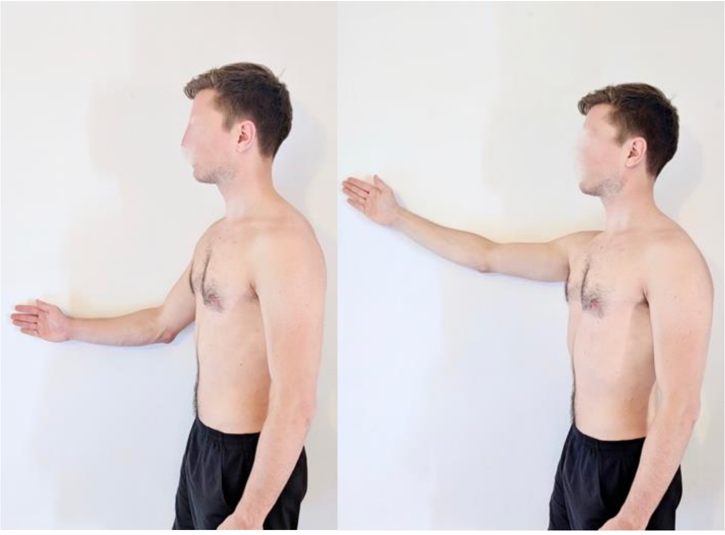

Exercise 1.2: Two-handed axial loaded elevation

- Starting position Patient stands in front of the wall, feet in line with the hip joints, hand palms touching the wall and positioned in line with the shoulder joints
- Exercise: Slowly sliding the hands up the wall, 3x15- 20 repetitions
- Note: Pain free motion, upright posture without increasing lumbar lordosis
- Progression: Variable support for the hands (pillow/ball)

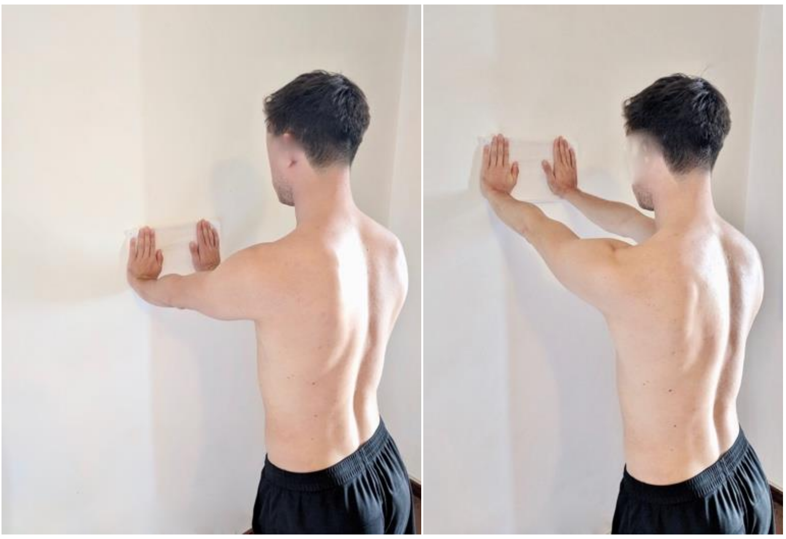

Exercise 1.3: Prone retroflexion ("I")

- Activation of the scapula stabilizers
- Starting position: prone position, elbows extended, thumbs in external rotation
- Exercise: Retroflexion and scapula retraction holding fully retracted position for 2-5 seconds, 3x15-20 repetitions

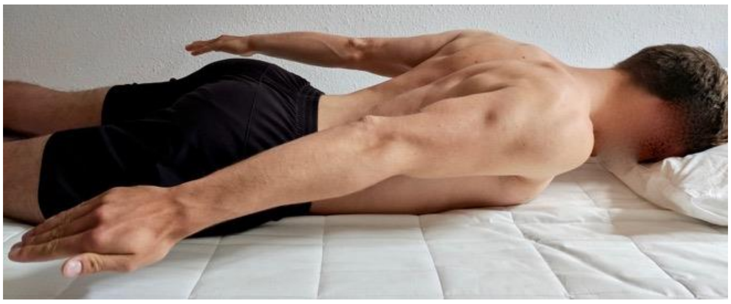

Exercise 1.4: Prone shoulder external rotation in 20° abduction ("W")

- Activation of the scapula stabilizers
- Starting position: prone position, elbows flexed with shoulder in 20°abduction
- Exercise: Raise arms and scapula retraction holding fully retracted position for 2-5 seconds, 3x15-20 repetitions

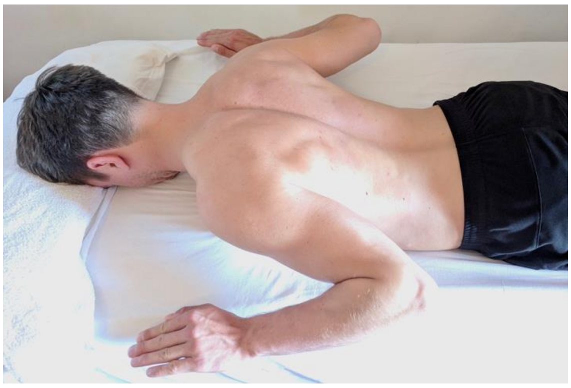

Exercise 1.5: Isometric external rotation

- Strengthening and activation of external rotators
- Starting position: Elbows in 90° flexion, shoulder and hand in neutral position
- Exercise: Hold isometric external rotation against a resistance band/Wall for 5-10 seconds, 3x15-20 repetitions

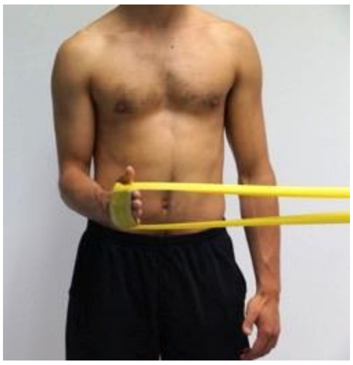

Level II:

Exercise 2.1: Elevation with resistance band

- Starting position: Patient stands with the back facing the wall, feet positioned in line with hip joints, knees slightly flexed back and head are leaning against the wall, resistance band in both hands, thumbs point up
- Exercise: Head and back are pushed towards the wall, neck is extended, active neutralization of lumbar lordosis. Flexion of both arms with simultaneous external rotation against a resistance band whilst sustaining core body tension, 3x15- 20 repetitions
- Note: Start to 90° flexion and progress to full ROM

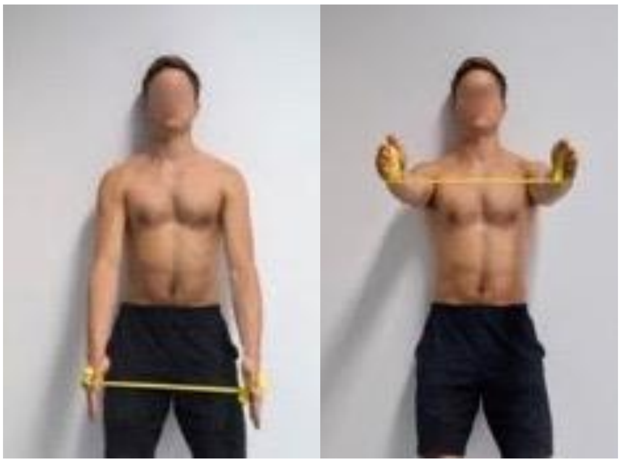

Exercise 2.2: Prone horizontal external rotation ("L")

- Activation of the scapula stabilizers
- Starting position: prone position, shoulder in 90° flexion and abduction, elbows 90° flexed, thumbs points upwards in external rotation
- Exercise: External rotation and scapula retraction holding fully retracted position for 2-5 seconds, 3x15-20 repetitions

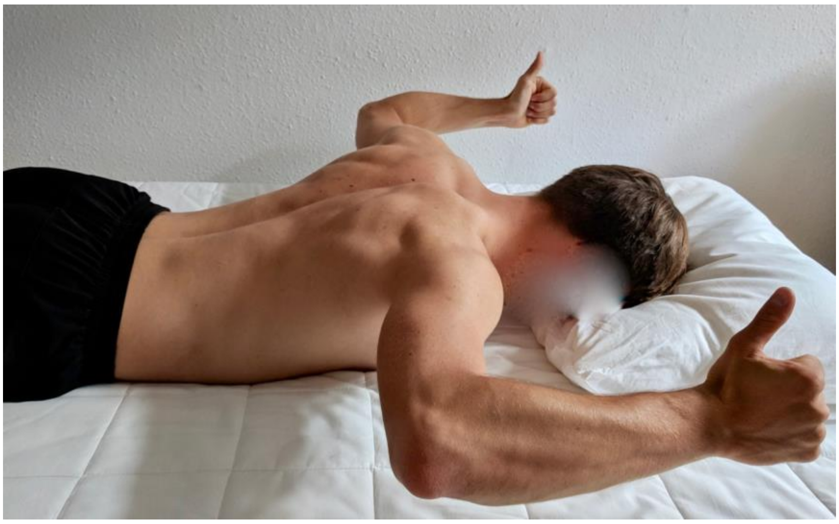

Exercise 2.3: Prone horizontal abduction and external rotation ("T")

- Activation of the scapula stabilizers

- Starting position: prone position, shoulder in 90° flexion and abduction, elbows extended, thumbs points upwards in external rotation
- Exercise: Raise arms and scapula retraction holding fully retracted position for 2-5 seconds, 3x15-20 repetitions

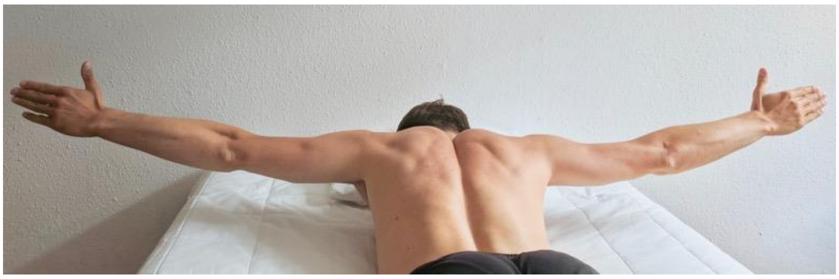

Exercise 2.4: Concentric external rotation with trunk rotation

- Concentric training of external rotators
- Starting position: Elbows in 90° flexion and hands neutral, shoulder in 20° internal rotation, resistance band in one hand
- Exercise: External rotation against a resistance band, 3x15-20 repetitions
- Progression: Starting position in 90° abduction

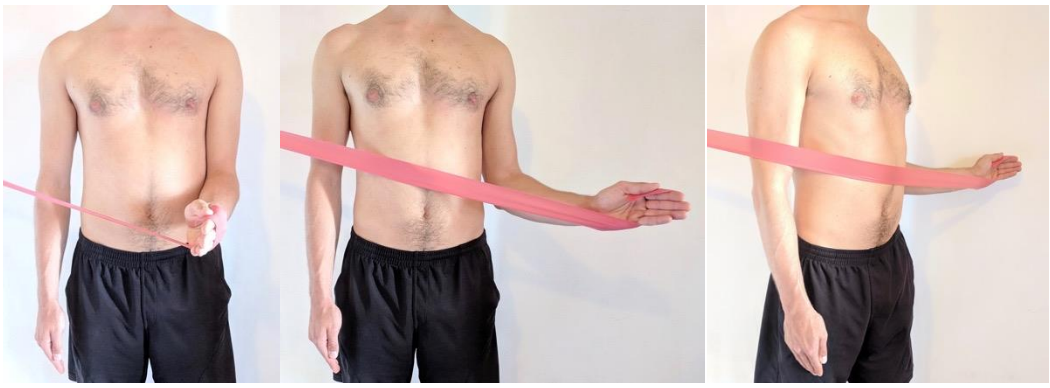

Exercise 2.5: Concentric external rotation in 90° abduction

- Concentric training of external rotators
- Starting position: Elbows in 90° flexion and abduction positioned on a stable surface, shoulder in 20° internal rotation, resistance band in one hand
- Exercise: External rotation against a resistance band, 3x15-20 repetitions

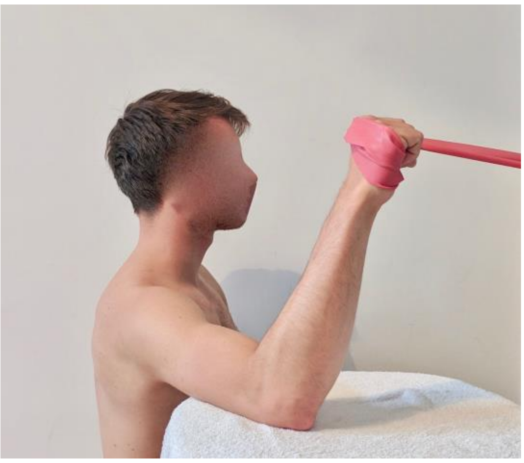

Exercise 2.6: Standing One-handed Row:

- Starting position: Elbows extended with one hand on a stable surface, resistance band in one hand with anchor at height of feet
- Exercise: Pull and scapula retraction ("elbow in back pocket") against a resistance band, 3x15- 20 repetitions

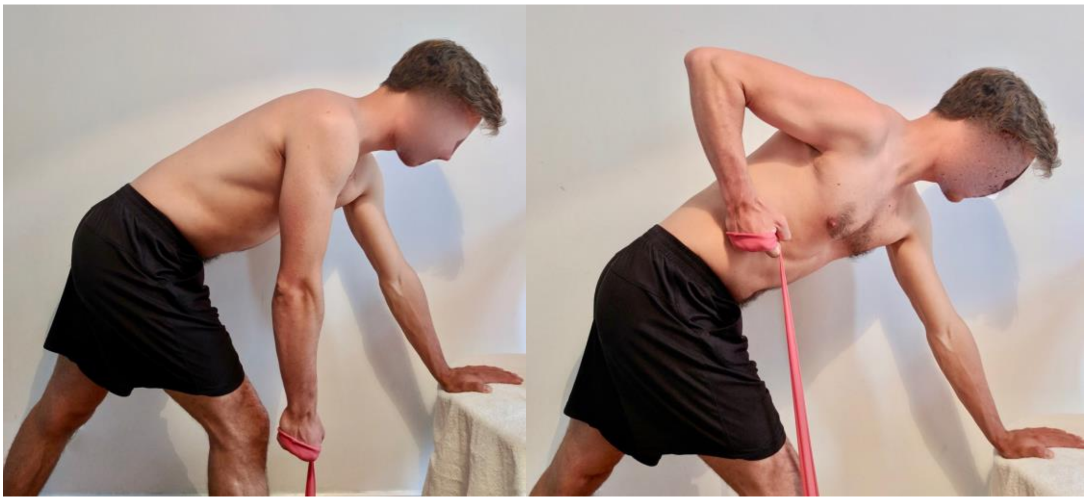

Exercise 2.7: Eccentric external rotation

- Eccentric training of external rotators
- Starting position: Elbows in 90° flexion and hands neutral, shoulder in external rotation, resistance band in one hand
- Exercise: Slowly rotate internally, 3x15- 20 repetitions

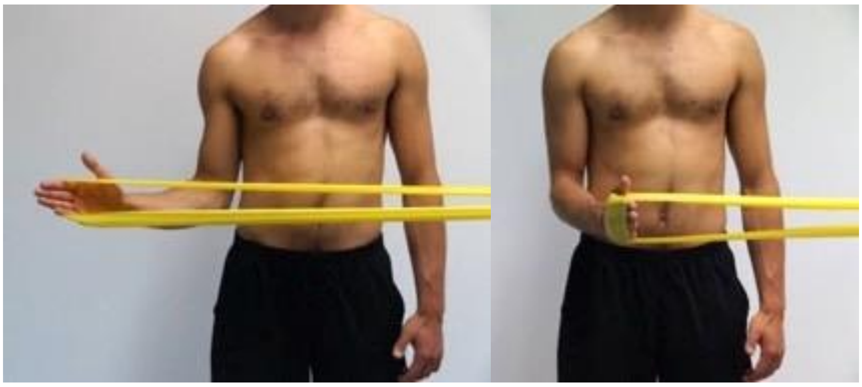

Exercise 2.8: Wall Scapular Push Up

- Starting position: Patient stands in front of the wall, feet in line with the hip joints, hand palms touching the wall and positioned in line with the shoulder joints, the scapulae are retracted
- Exercise: Push-ups against the wall by means of shoulder protraction, 3x15- 20 repetitions
- Note: Elbows extended and pointing towards the ground
- Progression: Unstable surface

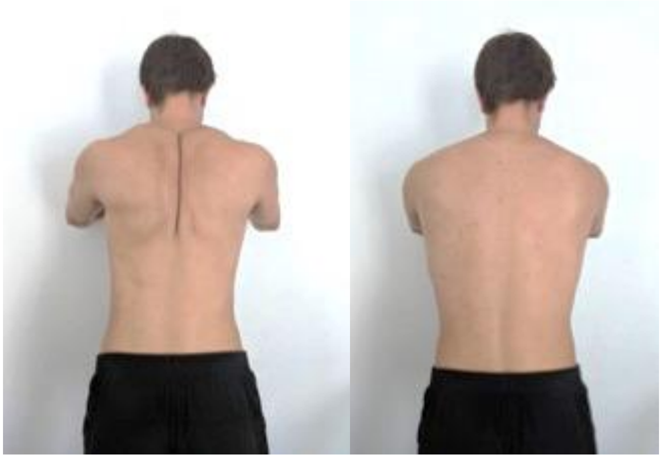

Exercise 2.9: Ball Wall Circles

- Starting position: Patient holds a ball in the hand of the affected arm and leans against a wall in 90° abduction
- Exercise: Lean body towards the wall whilst stabilizing the position of the hand on the ball, perform small circle motions, 3x15- 20 repetitions

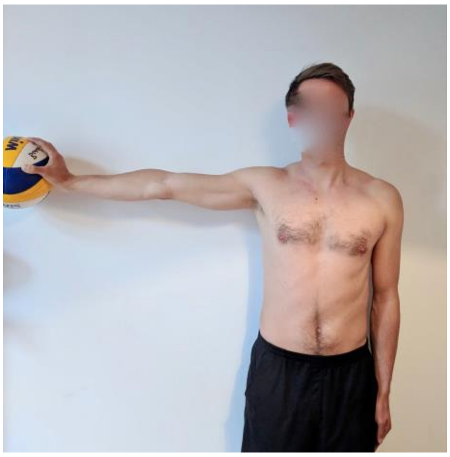

Exercise 2.10: Contralateral arm raises with ipsilateral leg raise

- Starting position: Patient holds a ball in the hand of the affected arm and leans against a wall
- Exercise: The contralateral arm is raised, whilst stabilizing the position of the hand on the ball with the ipsilateral leg raised, 3x15- 20 repetitions
- Note: Activate hip/core in diagonal pattern

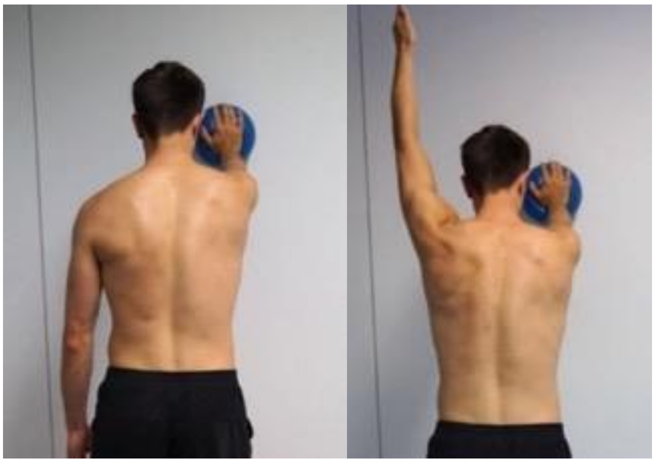

Level III:

Exercise 3.1: Pylometric exercises (two handed chest pass/overhead throwing)

- Start with two handed exercises, 3x15- 20 repetitions
- Progression: Increased weighted medicine ball, faster and higher repetitions

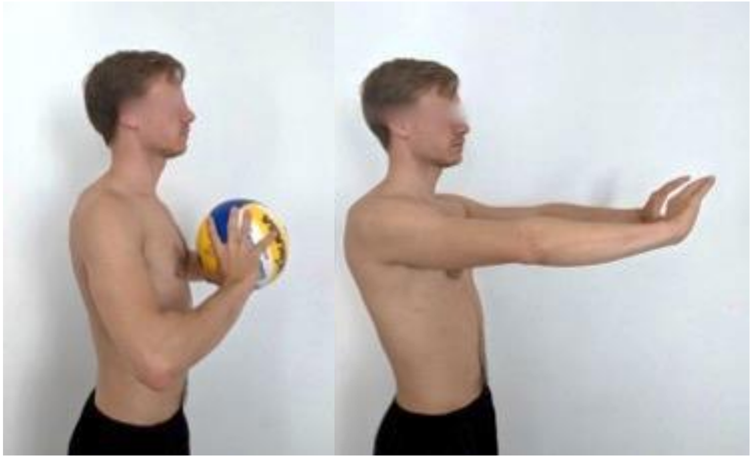

Exercise 3.2: Patient oriented sports training (POST)

- Complex movement exercises (e.g. one handed throwing, boxing, dribbling)
- Progression: Increased repetitions and complexity

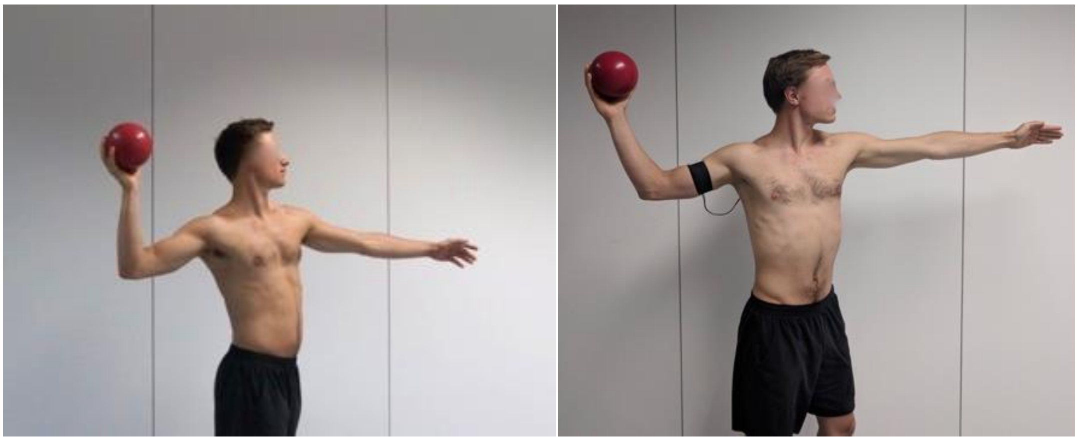

Exercise 3.3: Reverse Butterfly

- Starting position: Arms in 90° abduction and 30° horizontal-flexion, elbows extended, thumbs point upwards, resistance band in both hands
- Exercise: Bilateral horizontal extension of both arms and scapula retraction against a resistance band, 3x15- 20 repetitions

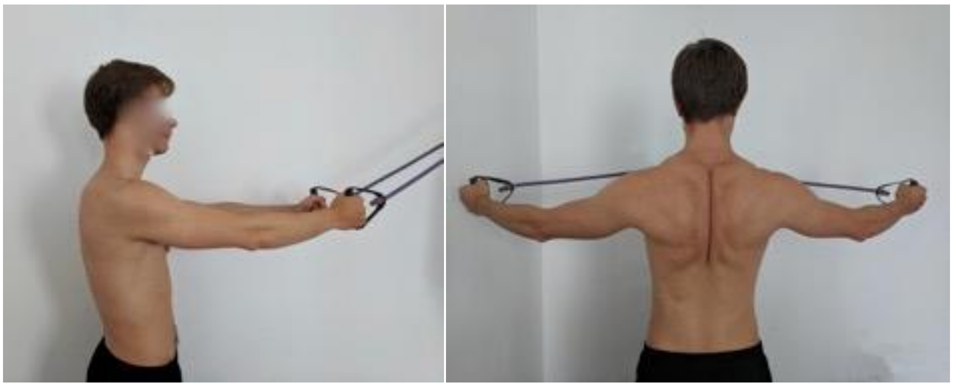

Exercise 3.4: Concentric low row

- Starting position: feet positioned in line with hip joints, hips/trunk flexed, resistance band in both hands with anchor at height of feet
- Exercise: Concentric low row motion with hips/trunk extension and scapula retraction, 3x15-20 repetitions

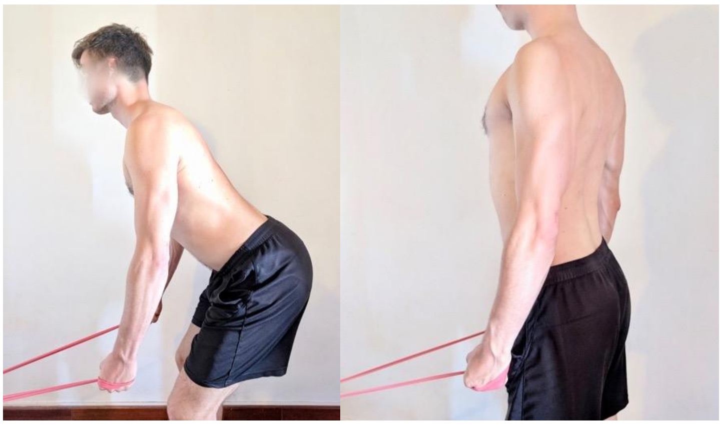

Exercise 3.5: Diagonal external rotation

- Starting position: Elbows in 90° flexion and hands neutral, shoulder in 20° internal rotation, resistance band in one hand
- Exercise: Slowly rotate diagonal externally to reach 90° flexion and abduction with additional external trunk rotation, 3x15- 20 repetitions

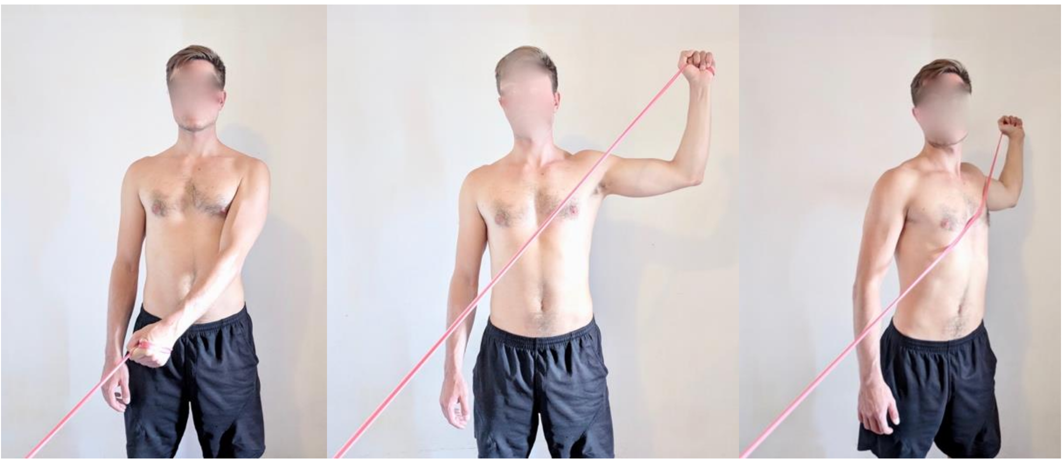

Exercise 3.6: Scapular Push Up

- Starting position: Push-up position, palms in line with the shoulder joints, shoulder retracted
- Exercise: Push-ups with scapula protraction, 3x15- 20 repetitions
- Note: Elbows extended pointing towards the floor
- Progression: Unstable surface

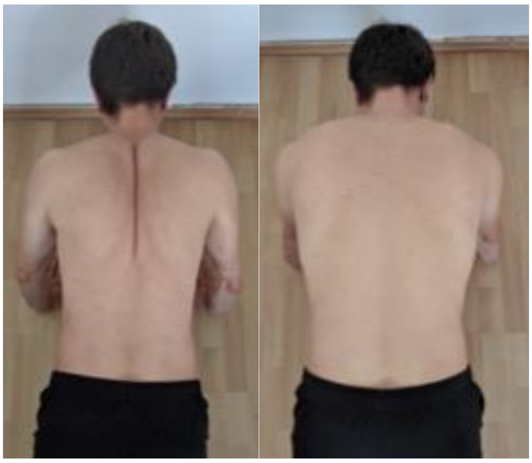

Supplementary Table. Comparison of the baseline characteristics of patients with and without an improvement of the Western Ontario Shoulder Instability Index (WOSI) in the neuromuscular electrical stimulation enhanced physiotherapy (NMES-PT) group that exceeds the minimally clinically important difference (MCID) of 10.4 % points.

	**Change in WOSI <MCID (n=10)**	**Change in WOSI >MCID (n=14)**	**p-value**
Age, years<br>(mean + standard deviation)	20.6±4.0	19.2±4.8	0.480
Female gender	30%	64%	0.214
Dominant side affected	60%	64%	1.000
Body Mass Index, kg/m^2<br>(mean + standard deviation)	23.8±3.3	21.8±2.7	0.101
Bilateral affection	60%	36%	0.408
Sulcus Sign<br>- Grade 0<br>- Grade 1<br>- Grade 2	 40%<br>50%<br>10%	 36%<br>64%<br>0%	0.660
Beighton Score<br>- 0-2 points<br>- 3-4 points<br>- 5-9 points	 40%<br>30%<br>30%	 36%<br>36%<br>29%	1.000
Scapular Dyskinesis	80%	86%	1.000
Other health problems	20%	29%	1.000
